# Dynamic dysregulation of transcriptomic networks in brainstem autonomic nuclei during hypertension development in the female spontaneously hypertensive rat

**DOI:** 10.1152/physiolgenomics.00073.2023

**Published:** 2023-12-25

**Authors:** Alison Moss, Lakshmi Kuttippurathu, Ankita Srivastava, James S. Schwaber, Rajanikanth Vadigepalli

**Affiliations:** Daniel Baugh Institute for Functional Genomics and Computational Biology, Department of Pathology and Genomic Medicine, Thomas Jefferson University, Philadelphia, Pennsylvania, United States

**Keywords:** autonomic control circuits, brainstem, neurogenic hypertension, spontaneously hypertensive rat, transcriptomics

## Abstract

Neurogenic hypertension stems from an imbalance in autonomic function that shifts the central cardiovascular control circuits toward a state of dysfunction. Using the female spontaneously hypertensive rat and the normotensive Wistar–Kyoto rat model, we compared the transcriptomic changes in three autonomic nuclei in the brainstem, nucleus of the solitary tract (NTS), caudal ventrolateral medulla, and rostral ventrolateral medulla (RVLM) in a time series at 8, 10, 12, 16, and 24 wk of age, spanning the prehypertensive stage through extended chronic hypertension. RNA-sequencing data were analyzed using an unbiased, dynamic pattern-based approach that uncovered dominant and several subtle differential gene regulatory signatures. Our results showed a persistent dysregulation across all three autonomic nuclei regardless of the stage of hypertension development as well as a cascade of transient dysregulation beginning in the RVLM at the prehypertensive stage that shifts toward the NTS at the hypertension onset. Genes that were persistently dysregulated were heavily enriched for immunological processes such as antigen processing and presentation, the adaptive immune response, and the complement system. Genes with transient dysregulation were also largely region-specific and were annotated for processes that influence neuronal excitability such as synaptic vesicle release, neurotransmitter transport, and an array of neuropeptides and ion channels. Our results demonstrate that neurogenic hypertension is characterized by brainstem region-specific transcriptomic changes that are highly dynamic with significant gene regulatory changes occurring at the hypertension onset as a key time window for dysregulation of homeostatic processes across the autonomic control circuits.

**NEW & NOTEWORTHY** Hypertension is a major disease and is the primary risk factor for cardiovascular complications and stroke. The gene expression changes in the central nervous system circuits driving hypertension are understudied. Here, we show that coordinated and region-specific gene expression changes occur in the brainstem autonomic circuits over time during the development of a high blood pressure phenotype in a rat model of human essential hypertension.

## Graphical abstract

**Figure:**
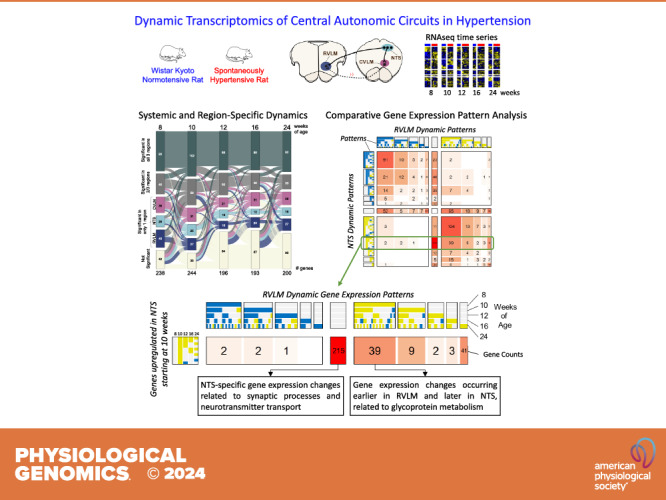

## INTRODUCTION

Hypertension is a major disease and is the primary risk factor for cardiovascular complications and stroke. The pathophysiology of essential hypertension is highly complex with much evidence pointing to high blood pressure being a polygenic trait (1). Neurogenic hypertension is a subtype of essential hypertension where the development is primarily driven by abnormalities in the sympathetic output from the higher autonomic centers to the rest of the cardiac control circuitry, which can lead to pathophysiological consequences (2–4). Given the genetic basis of hypertension, it is crucial to understand the underlying changes throughout the developmental process. The spontaneous hypertensive rat (SHR) is one of the most successful inbred animal genetic models for studying hypertension (5–13). The SHR genome has been sequenced and the physiology has been studied extensively (11, 14). The SHR background has been used in recombinant inbred models to further interrogate the genetics of hypertension (12). However, the system-wide molecular characteristics of the brainstem circuits have been explored in a very limited way and studies have yet to analyze and compare the transcriptomic profiles between autonomic control regions of the brainstem.

The background activity of the large groups of sympathetic efferents that has major implications in blood pressure regulation is governed by neurons residing in the nucleus of the solitary tract (NTS), rostral ventrolateral medulla (RVLM), and caudal ventrolateral medulla (CVLM) (2, 15–17). The NTS is the main processing center, receiving inputs from other autonomic centers as well as both sympathetic and parasympathetic afferents. The NTS projects to both the CVLM and RVLM, where the RVLM is the site of excitatory output from the brainstem to the spinal cord, whereas the main function of the CVLM is to inhibit or dampen the excitatory activity of the RVLM (4, 15, 16, 18–23). Together, these brainstem regions coordinate the sympathetic and parasympathetic reflexes and play a critical role in neurogenic hypertension (4, 15, 17).

There have been a limited number of studies exploring the transcriptomics of the brainstem circuits in hypertension. To date, no study has examined transcriptomic profiles of specific autonomic centers during the development of hypertension. Several studies have focused on a specific pathway or process including gene expression analysis. For example, the role of inflammatory factors in the NTS in the maintenance of hypertension in SHRs was analyzed through cytokine profiling (1, 24–28). Other studies have quantified genes involved in catecholaminergic homeostasis in multiple organs and their impact on blood pressure control (29–31). There has been some comparative RNA-sequencing (RNA-seq) analysis of brainstem transcriptomes in the hypertensive inherited stress-induced arterial hypertension (ISIAH) compared with normotensive Wistar Albino Glaxo male rats (32) and newborn SHR versus Wistar–Kyoto (WKY) rats (33). These studies analyzed the brainstem as a whole and hence lacked the resolution of individual regions in the autonomic control circuit. In addition, the analysis is typically performed after a stable hypertension phenotype has been established, thus missing an opportunity to evaluate the dynamic components of the development of the phenotype. Previous work from our group examined the dynamics of global microRNA regulation in the male SHR, with accompanying time series gene expression data at a pathway scale (34). In this previous study, we found a novel regulatory network motif consisting of microRNAs in upregulated SHRs likely downregulating inhibitors of prohypertensive processes such as angiotensin II signaling and leukotriene-based inflammation in the NTS. Previous studies have shown RVLM-enhanced angiotensinergic activation and reduced GABAergic inhibition as contributing to hypertension in SHRs (35–37). Our previous results on the lower levels of microRNAs in the RVLM are consistent with putative microRNA-mediated regulation of these processes in the RVLM (34).

In the present study, we carried out an integrative time series analysis of transcriptome-wide changes in multiple brainstem regions contributing to hypertension development in female SHRs. We used a dynamic pattern analysis to identify the differentially expressed genes (DEGs) and exhaustively examined the similarities and differences in transcriptomic changes between the nuclei using a pairwise comparative pattern analysis. The pattern analysis and comparisons enabled us to uncover both dominant as well as subtle changes in gene expression within and between regions over time, pointing out key differentially regulated processes and systems in an unbiased manner. Here, we report widespread systemic changes that occur throughout the brainstem regardless of the stage of hypertension development. Beneath this strain-dependent signature is a cascade of differential expression that propagates throughout the brainstem, starting in the RVLM at the prehypertension stage at 8 wk of age and shifting to the NTS during 10 and 12 wk, corresponding to the onset of hypertension. We further examined the transcriptomic dynamics of pathways contributing to neuronal excitability as well as cholinergic and catecholaminergic biosynthesis processes throughout the brainstem.

## MATERIALS AND METHODS

### Animal Model

Female WKY (WKY/NHsd) rats (control) and SHRs (SHR/NHsd) obtained from Envigo were housed one per cage in the Thomas Jefferson University animal facility to avoid animal-to-animal stress from dominance that could affect blood pressure. We obtained samples from *n* = 3–6 animals per strain per timepoint (specifically, for WKY rats, *n* = 5, 4, 3, 3, and 3, and for SHRs, *n* = 5, 5, 6, 6, and 6 for 8, 10, 12, 16, and 24 wk of age, respectively). Facilities were maintained at constant temperature and humidity with 12:12-h light-dark cycles (lights on at zeitgeber time 0). All animal treatment protocols were approved by the Thomas Jefferson University Institutional Animal Care and Use Committee. Blood pressure was recorded at 12 and 24 wk of age in all of the corresponding animals using the CODA mouse rat tail-cuff system from Kent Scientific, following our previously published approach (34).

### Tissue Collection

Samples were collected across five timepoints throughout the development of hypertension. Eight weeks is the age at which SHR animals are considered prehypertensive. The age of 10–12 wk, considered the onset period of hypertension, was of particular interest, and animal tissues were collected at both timepoints. Animals collected at 16 and 24 wk of age correspond to the full hypertensive state and extended chronic hypertensive state, respectively. Each animal was euthanized by rapid decapitation that was preceded by 5% isoflurane in O_2_. The brain was quickly removed and placed into the ice-cold artificial cerebrospinal fluid for separation of the brainstem, which was then rapidly frozen in an optimal cutting temperature (OCT) medium for cryosectioning. No more than 10 min passed between decapitations and freezing the tissue in OCT for each animal. Brainstems were sectioned at 200 μm, and the regions of interest were extracted using a tissue punch 1 mm in diameter (Stoelting). We followed the same approach we have previously used to obtain consistent and atlas-guided samples of the relevant brainstem autonomic control regions (34, 38, 39). Briefly, we used the *Paxinos and Watson Rat Brain Atlas* to guide punches that were matched to the size of the nucleus and rostral-caudal level and specifically to the *XY* position of the dorsal vagal complex within the complex anatomy of the brainstem. After micropunching, the remnant sections were visually examined under a light microscope to confirm the acquisition of the relevant regions. The neuroanatomic features of the dorsal medulla at these rostral-caudal levels are highly differentiated and distinct, and the dorsal vagal complex (DVC) is easily delineated from surrounding structures. Bilateral region punches from one animal were treated as a single sample for transcriptomic analysis.

### RNA Extraction and Sequencing

Total RNA was extracted using the Direct-Zol microprep kit from Zymo Research and stored at −80°C. All samples had an absorbance at 260/280 nm of >1.8. DNase treatment was performed as part of the microprep protocol. Concentration and integrity were assessed with ND-1000 (NanoDrop, Philadelphia, PA). Total RNA was sent to the GENEWIZ transcriptomics service for quality control, library preparation, and subsequent RNA-seq (GENEWIZ, South Plainfield, NJ). An average of 22 million reads were generated per sample. Paired-end raw fastq files were mapped to the Rat Genome Sequencing Consortium (RGSC) rat (rn6-RGSC) reference genome with the use of the RSEM 1.3.1 STAR (version 2.6.1) aligner, with default parameters (40). The transcript abundance was subsequently quantified using the RSEM pipeline.

### Data Processing Differential Expression Analysis

The first step in the data processing was to adjust for any batch effects. This was accomplished using ComBat_seq through the “sva” package in R (version 3.40.0) (41). The resulting count data was used to construct a DESeq data set. Before differential expression analysis, the raw count data in the DESeq data set was filtered to remove genes with low expression. Count data were normalized using the regularized log transform (rlog) from the DESeq2 package. Genes with a maximum expression value of <3.5 were filtered out and not considered for downstream statistical analysis. The threshold of 3.5 was chosen by comparing the raw counts to the normalized values and ensuring that all genes with fewer than 50 total raw counts were filtered out. Differential expression analysis was run on the filtered data set using DESeq software (42). We built a DESeq model based on age, strain, and strain:age interaction. Log_2_(fold change) and *P* values were calculated using the Wald test to compare strains at each timepoint and to compare fluctuations in expression for each strain compared with its expression at the initial timepoint of 8 wk. *P* values from the above method were subjected to a multiple testing correction using the “qvalue” package in R (version 2.24.0) (43). Log_2_(fold change) and the accompanying *q* values were used for downstream analysis. For visualization of relative expression levels, genes were *z*-scored across ages and strains for each region separately. For initial differential expression analysis, genes with log_2_(fold change) > 2 and *q* < 0.01 were used for downstream pathway analysis. We included fewer WKY animals in the 12, 16, and 24 wk of age groups to reduce the number of animals used while still allowing for statistical analysis. Our results show consistent gene expression patterns within the WKY animals at each age and a significant number of DEGs at a strict *q* value threshold, supporting our design for reduced animal usage.

### Dynamic Pattern Analysis Using COMPACT

The differential expression shift in genes at each timepoint for SHRs was calculated relative to WKY rats. Genes with *q* < 0.05 were considered for analysis, and a fold change cutoff of 1.5 [log_2_(fold change) > 0.585] was used to discretize the data into three levels (+1, 0, −1), where +1 indicates upregulation, 0 indicates no change in expression, and −1 indicates downregulation. This approach results in each gene having three five-digit pattern codes, one for each region of the brainstem, with a possible 243 patterns for each gene profile (3 discretizations, 5 timepoints, 35 = 243). It should be noted that for the purpose of broad pattern analysis, numerical codes were assigned based only on fold change and not on the accompanying *q* value. For example, pairs of regions were compared, and the total numbers of genes for each possible combination (243 × 243) of expression patterns between two regions were counted. The resulting COMPACT matrix can be broadly segmented into clusters that define a particular pattern-pattern association between two brainstem regions. For example, genes in the upper right and lower left quadrants represent genes that are oppositely dysregulated in the two regions of interest, while the upper left and lower right quadrants represent genes that are similarly dysregulated in both regions, and the center row and column represent genes that are dysregulated in one region and not the other. This method was also used to compare all three regions by extracting the intersection of significant gene groups between COMPACT patterns.

### Pathway Enrichment Analysis

The dynamic patterns were analyzed for three gene ontologies (molecular function, biological process, and cellular component) using DAVID (44). Enrichment analysis using Gene Ontology of the DEGs was performed using the R package ClusterProfiler (version 4.0.2) (45, 46). *Rattus norvegicus* was used as the background reference for the overrepresentation analysis.

### Availability of Supporting Data/Additional Files

The time series RNA-seq paired-end data were deposited in the National Center for Biotechnology Information Gene Expression Omnibus and Sequence Read Archive under Accession No. GSE234784.

## RESULTS

### Dynamic Differential Expression During Hypertension Development

**Figure 1.**
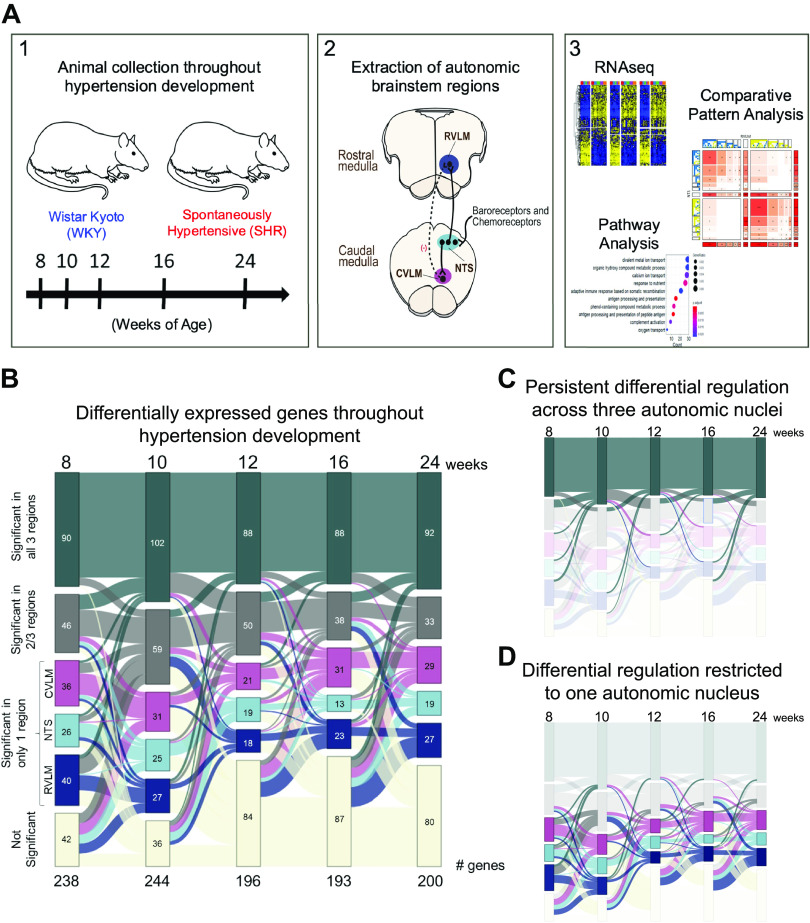
Time series transcriptomic analysis of brainstem autonomic control circuits during the development of hypertension. A: workflow and overview of the analysis methods. *1*) Brainstems from female spontaneously hypertensive rats (SHRs) and Wistar–Kyoto (WKY) rats were collected throughout the development of hypertension at 8, 10, 12, 16, and 24 wk of age. *2*) For each brainstem, tissue from the nucleus of the solitary tract (NTS), caudal ventrolateral medulla (CVLM), and rostral ventrolateral medulla (RVLM) were excised, and RNA was extracted. *3*) The resulting RNA-sequencing data were analyzed for differential expression, pathway enrichment, and comparative dynamic patterns across brainstem regions. *n* = 3–6. For WKY rats, *n* = 5, 4, 3, 3, and 3; for SHRs, *n* = 5, 5, 6, 6, and 6 for 8, 10, 12, 16, and 24 wk of age, respectively. *B*: Sankey plot of the 280 genes showing differential expression at more than one timepoint (*q* < 0.01, fold change > 2). The diagram shows the number of genes significant in one or more regions at each timepoint with the flow from left to right indicating in which regions the genes were differentially expressed over time. Numbers along the bottom indicate how many of the 280 genes with dynamic differential expression showed statistically significant differential expression at the timepoint of interest. For each node, the number of genes significant in the corresponding region(s) at each timepoint is shown. Bands between timepoints are colored according to their target node, and their designation at the following timepoint. *C*: genes that were differentially expressed in one or more regions were largely dysregulated persistently over time, as indicated by a consistent width of the band across the timepoints. *D*: a cohort of genes was dysregulated in only one region at each stage of hypertension development. Some of these genes showed differential expression in multiple nuclei at certain timepoints (such as 10 wk of age) and returned to dysregulation in a single nucleus at later timepoints.

We sought to examine the dynamic and temporal changes occurring in three different brainstem autonomic nuclei throughout the development of hypertension in the female SHR and contrast these against the age-matched gene expression changes in the female WKY rat. The NTS, RVLM, and CVLM were excised from the brainstems of female SHRs and normotensive WKY rats. Bilateral tissue punches were collected (*n* = 3–6) at 8, 10, 12, 16, and 24 wk of age, corresponding to the stages considered to span the onset of hypertension in the SHR through to a state of extended chronic hypertension (Fig. 1*A*; 13, 34, 47). The mean arterial pressure (MAP) of WKY rats and SHRs at 12 wk of age was 122.245 ± 11.93 and 173.29 ± 24.6 mmHg, respectively. At 24 wk of age, MAPs of WKY rats and SHRs were 124.04 ± 5.56 and 186.42 ± 14.23 mmHg, respectively. The evolution of the hypertensive state seen in SHRs compared with WKY rats and the increase in blood pressure in SHRs between 12 and 24 wk are consistent with what is expected in the SHR model based on previous reports (34, 48). Analysis of RNA-seq time series data revealed that of the 14,270 genes with robust expression in at least one of the three brainstem regions, 1,531 genes showed statistically significant differential expression between SHRs and WKY rats at one or more timepoints (adjusted *q* value ≤ 0.01). Further filtering these genes for those with a SHR versus WKY fold change of ≥2 yielded 603 genes, of which 280 genes showed differential expression at more than one timepoint (Fig. 1*B*).

We sought to characterize the dynamic nature of these changes as well as their specificity to a brainstem nucleus. A subset of the 280 genes was differentially expressed at any given timepoint (varying between 193 and 244 genes; Fig. 1*B*). A dominant pattern with most genes was a persistent differential regulation between SHRs and WKY rats across all the timepoints in all three regions (Fig. 1*C*), indicating a system-wide shift in gene expression in SHR compared with WKY autonomic nuclei. Another feature of the data was that a subset of genes showed differential regulation only in one autonomic nucleus at any given timepoint (Fig. 1*D*). Pathway enrichment analysis of the 280 gene set showed a strong enrichment for immunological processes such as antigen processing and presentation (*q* < 0.001), activation of the complement pathway (*q* < 0.003), and the adaptive immune response (*q* < 0.005). Some notable genes in these processes are rat isoforms of the human major histocompatibility complex (MHC), which, in addition to their role in the immune response, have been shown to influence the excitability of neurons, specifically influencing NMDA receptor function and trafficking of AMPA receptors (49–51). Other enriched processes with an established influence on hypertension are those involved in oxygen binding and transport and hydrogen peroxide metabolic processes leading to oxidative stress (*q* < 0.01; 52, 53).

### Region-Specific Transient Differential Expression During Hypertension Development

**Figure 2.**
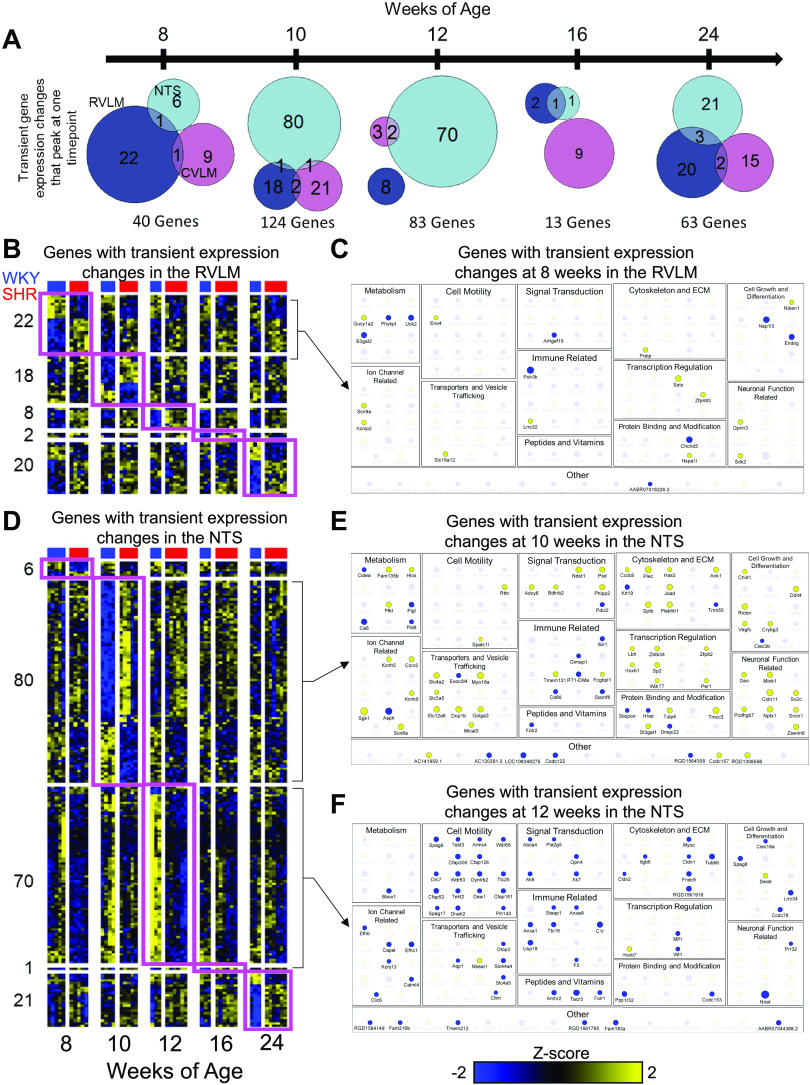
Highly transient differential gene expression in spontaneously hypertensive rats (SHRs) is specific to a brainstem region. *A*: the 603 differentially regulated genes (*q* < 0.01 and fold change > 2), 323 genes showed differential expression at only one of the five timepoints. Venn diagrams detail the regional distribution of these transient differentially expressed genes, revealing that the majority were specific to one brainstem region of interest. *B* and *D*: heatmap of all genes with highly transient differential expression in the rostral ventrolateral medulla (RVLM) (*B*) and nucleus of the solitary tract (NTS) (*D*). Genes are represented by rows, while each column is indicative of one sample replicate. Pink boxes indicate the timepoints at which the select genes were differentially expressed at or above the indicated threshold. *C*, *E*, and *F*: genes with transient expression changes at 8 wk in the RVLM (*C*) were categorized along with the genes transiently dysregulated in the NTS (*E* and *F*) based on their annotation to highlight the processes that were particularly dysregulated in each group. The size of the points was correlated to the overall abundance, with larger points indicating higher expression levels. Blue indicates downregulation; yellow indicates upregulation.

In addition to persistent and systemic regulation (Fig. 1*B*), a significant number of genes were transiently differentially expressed over time and these were largely region-specific (323 out of 603 genes; Fig. 2*A*). The early onset timepoint of 10 wk showed the largest number of transient DEGs (124 genes; Fig. 2*A*). In contrast, a set of 13 genes were differentially regulated only at 16 wk. The majority of the gene expression changes at 16 wk were not unique to that timepoint (Fig. 1*B*). Although the genes with differential expression at multiple timepoints were significantly regulated in more than one region (Fig. 1*B*), transient changes at only one of the timepoints were restricted to a single region (Fig. 2*A*). At 8 wk of age, gene expression changes in the RVLM dominate over those in the NTS and CVLM. At 10 and 12 wk, the gene expression changes in the NTS far outnumbered the changes in the other two regions. It was only at 16 wk that the CVLM showed a larger number of DEGs than the other two regions, but the number of genes regulated at this timepoint was significantly lower. Interestingly, at later stages of extended chronic hypertension, there appeared to be more of a balance in terms of the number of DEGs between the three regions (Fig. 2*A*).

At 8 wk of age, genes participating in a variety of neuronal functions were differentially expressed in RVLM, with notable ion channels such as *Scn9a* and *Kcnip2* being upregulated (Fig. 2, *B* and *C*). Increased expression of the Na^+^ channel *Scn9a* as well as the regulator of neurite outgrowth *Gprin3* indicated an increase in neuronal excitability (54), whereas the sidekick adhesion molecule *Sdk2* was localized to the synapse and contributed to synaptic connectivity (55). The patterns in the NTS at 10 and 12 wk were much more distinct (Fig. 2, *A* and *D*). At the onset of hypertension at 10 wk, 80 genes showed NTS-specific dysregulation including several ion channels and genes involved in transport, cell junction, and vesicle trafficking as well as neuronally related genes and transcription regulators (Fig. 2, *D* and *E*). SGK1 is a glucocorticoid-induced kinase that stabilizes Na^+^ transporters and has been linked to hypertension in the kidney while potentially providing protection against ischemic injury in the brain through modulation of other ion channels (56, 57). Genes such as *Mical3*, a scaffold for vesicle trafficking, and the K^+^-Cl^-^ cotransporter *Slc12a6* are both important for neuronal development (58, 59). The transcription factor HOXB1 has been found to regulate the stress response in noradrenergic neurons and may have an association with the transcriptional repressor REST, which has been shown to promote a neurosecretory phenotype (60–62).

Interestingly, at 12 wk, nearly all of the DEGs were downregulated in SHRs compared with WKY rats. However, a closer examination revealed that the downregulation in SHRs was due to a lack of upregulation observed in WKY rats at the later timepoints (Fig. 2, *D* and *F*). A large number of these genes were annotated for functions involved with cell motility and cytoskeletal function and are crucial to the formation and function of cilia and flagella. Primary cilia in neurons have been shown to influence nonsynaptic signaling of neuropeptides and hormones and those in close proximity to brain ventricles, such as the NTS, may survey the extracellular milieu present in cerebrospinal fluid (63, 64). The lack of induction of these genes in the SHR at this critical state of hypertension onset indicates attenuation of the NTS response to extracellular signaling, likely contributing to autonomic dysfunction.

### Unbiased Comparative Pattern Count Analysis to Assess Dynamic Trends

**Figure 3.**
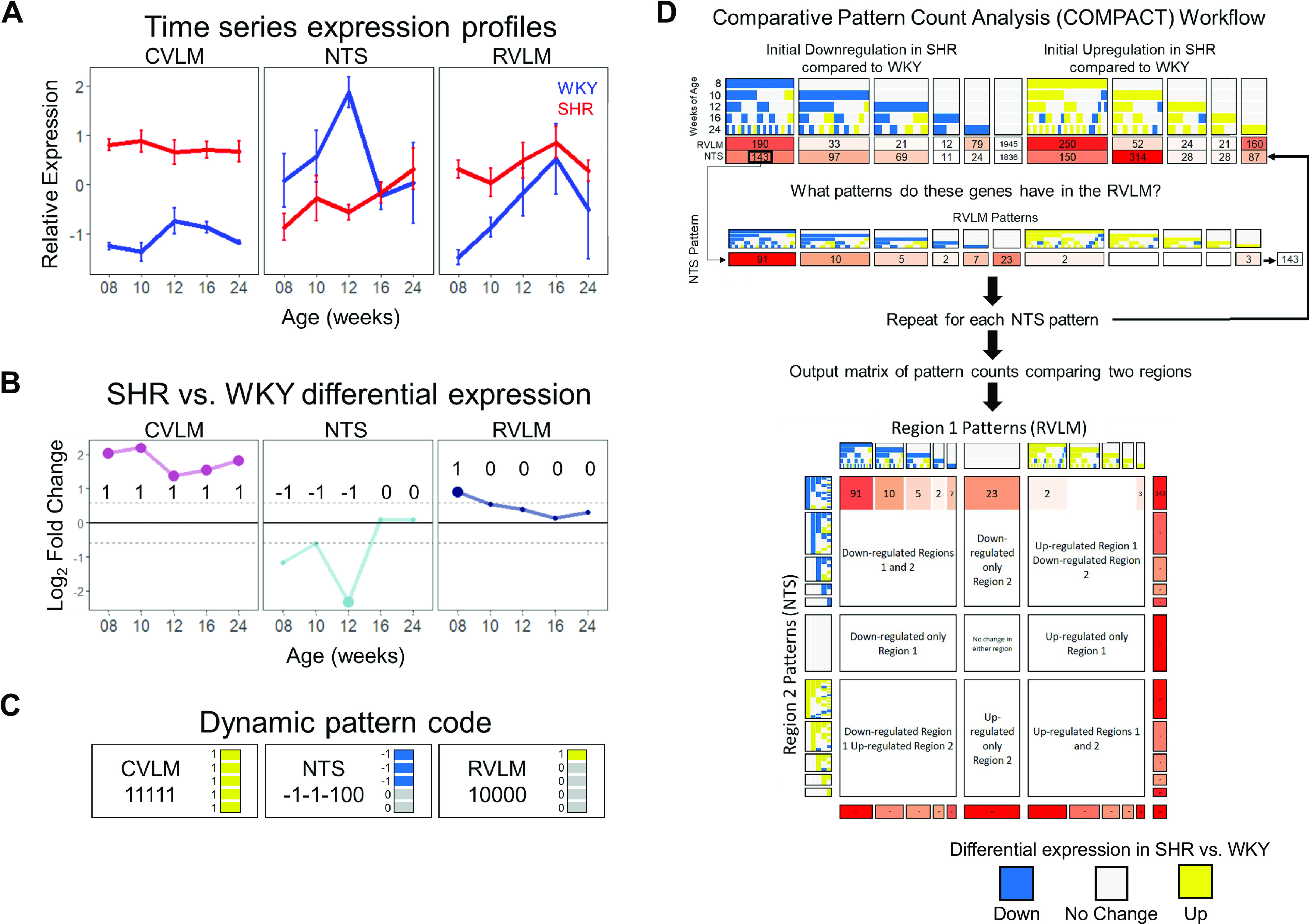
Comparative pattern analysis (COMPACT) workflow for unbiased analysis of dynamic differential expression data. *A*: illustrative gene expression profiles in the Wister–Kyoto (WKY) rat and spontaneously hypertensive rat (SHR) (representative gene *Igfbp5*). Normalized expression levels from *rlog* were transformed into a *Z* score for visualization. B: differential expression of gene profiles over time. Values represent the pairwise log_2_(fold change) (LFC) in SHRs compared with WKY rats at each timepoint. Enlarged points represent the statistical significance of *q* < 0.2. Dashed lines represent the threshold at which a gene was considered upregulated or downregulated in SHRs compared with WKY rats; in this case, LFC > 0.585 or fold change > 1.5. In this approach, each gene profile was given a numerical pattern code at each timepoint, where −1 represents downregulation, 1 represents upregulation, and 0 indicates no change in expression based on whether the differential expression at each timepoint fell below, above, or within the designated fold change cutoff at each timepoint, respectively. *C*: in the representative case of *Igfbp5*, in the caudal ventrolateral medulla (CVLM), there was upregulation beyond the designated threshold at all timepoints resulting in a pattern code of 11111. In the nucleus of the solitary tract (NTS), there was downregulation beyond the designated threshold at the first three timepoints with no dysregulation at the latter two timepoints resulting in a code of -1-1-100. The expression pattern in the rostral ventrolateral medulla (RVLM) yielded a code of 10000. For visualization of these codes in a larger COMPACT matrix, upregulation designated with “1” is shown in yellow, downregulation designated as “−1” is shown in blue, and no dysregulation designated by “0” is shown in gray. *D*: COMPACT patterns were grouped based on which timepoint they initially showed dysregulation. Examination of the number of genes in each pattern group for each brainstem region gave initial clues as to the overall transcriptomic changes in the region. For example, at 8 wk of age, there were 190 genes that were initially downregulated in the RVLM, whereas 143 genes were initially downregulated in the NTS. We further examined these 143 genes with initial downregulation at 8 wk in the NTS and compared the dynamic response patterns of these genes in the RVLM. By repeating this process for each pattern in one region, we were left with a matrix of counts that could describe the distribution of genes with similar and dissimilar expression patterns between two regions. The colored patterns, as described in *C*, are read from top to bottom (and left to right along the side). Upregulation is shown in yellow, downregulation in blue, and no dysregulation in gray.

We used an unbiased comparative pattern count analysis (COMPACT) workflow that highlights dynamic and subtle patterns in the transcriptomic data that are often overlooked in typical differential expression analysis (65–67). In this approach, each gene expression profile over time is transformed into a discrete pattern code based on thresholding SHR versus WKY fold changes at each timepoint (Fig. 3, *A–C*). The dynamic gene expression shift in the SHR is calculated relative to the WKY rat for each region. This results in a possible 243 patterns for each gene in each region (3 levels at each timepoint, 5 timepoints = 3^5^ permutations). These pattern codes are used in downstream COMPACT analysis to enumerate all possible changes in the pattern codes between regions to allow an exhaustive pairwise comparison of expression changes (Fig. 3*D*, *top*). To evaluate the comparative pairs of dynamic expression shifts between regions at multiple timepoints, the patterns present in one region can be compared with the pattern assignments of the same genes in another region resulting in a matrix comparing patterns between two regions (Fig. 3*D*). This approach enables a layered exploration of genes with common, novel, and altered differential expression patterns between regions, as illustrated in the next four sections.

### Comparing Dynamic Expression Patterns Between the NTS and RVLM

**Figure 4.**
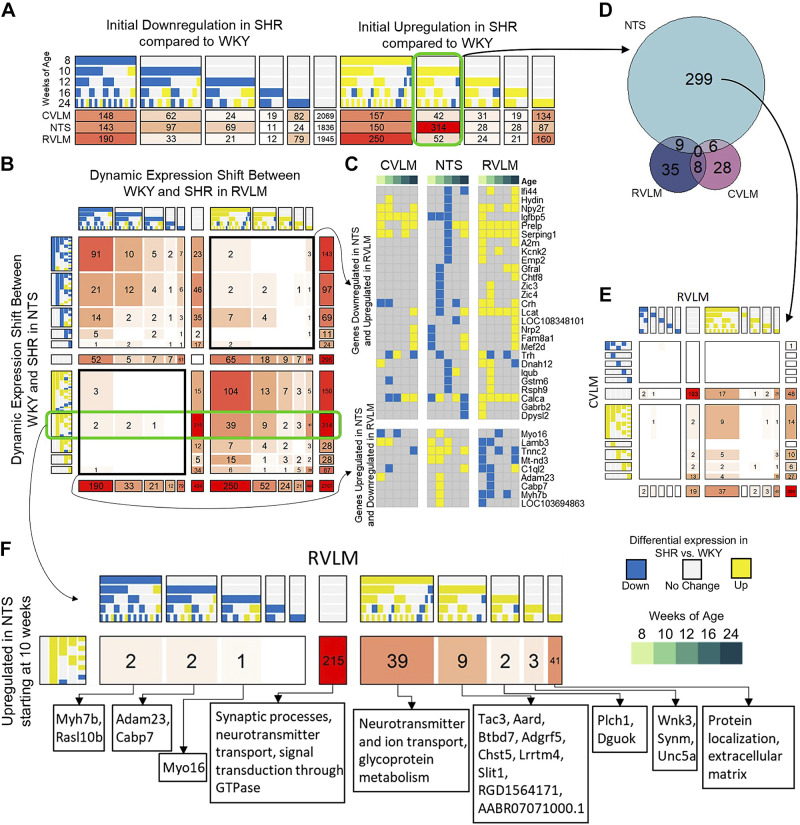
Comparative pattern analysis of spontaneously hypertensive rat (SHR) vs. Wistar–Kyoto (WKY) differential expression dynamics between the nucleus of the solitary tract (NTS) and rostral ventrolateral medulla (RVLM). *A*: pattern counts for each region were categorized by the stage of their initial up or downregulation. The cells of each row are colored based on the number of genes. *B*: COMPACT matrix comparing dynamic gene expression patterns in the NTS with those in the RVLM. Bolded boxes around the top right and bottom left quadrants represent areas of opposite regulation where genes are downregulated in the NTS and upregulated in the RVLM (*top right*) or vice versa (*bottom left*). These genes are then represented in the discretized heatmap in *C*. The elements of the matrix are colored based on the corresponding number of genes. *C*: heatmap showing the discretized patterns of genes that were initially downregulated in the NTS and upregulated in the RVLM (*top*) and initially upregulated in the NTS and downregulated in the RVLM (*bottom*). Each column is representative of the discretized patterns for the given region at each timepoint averaged over multiple biological replicates within each group. *D*: Venn diagram showing the overlap between genes that were initially upregulated at 10 wk of age across all three brainstem regions. *E*: COMPACT matrix examining the distribution in the caudal ventrolateral medulla (CVLM) and RVLM of the 299 genes that showed NTS-specific upregulation at 10 wk. *F*: zoomed-in view of the 314 genes that were initially upregulated in the NTS at 10 wk of age to illustrate the corresponding dynamics of these genes in the RVLM, along with the enriched processes.

We applied the COMPACT workflow to the genes differentially regulated at one or more timepoints for all regions (*q* < 0.05). Of the 243 possible patterns in each region, genes were distributed in 81 dynamic patterns (Fig. 4*A*). Dominant patterns in the RVLM and CVLM consisted of early up- and downregulation in SHRs versus WKY rats, starting at 8 wk, whereas the NTS also showed a significant number of genes (314) that showed upregulation beginning at 10 wk. There were far more genes dysregulated at the stage of chronic hypertension, 24 wk of age, in the RVLM and CVLM compared with the NTS (Fig. 4*A*).

We first compared the dynamic gene expression patterns in the NTS compared with those in the RVLM (Fig. 4*B*). A dominant feature evident in the COMPACT matrix is the region-specific differential regulation (Fig. 4*B*, 404 NTS-specific in the *middle* column and 295 RVLM-specific in the *middle* row). Several genes showed similar differential regulation in both regions (diagonal quadrants in Fig. 4*B*). A number of genes showed opposite patterns of regulation in SHR versus WKY rats (anti-diagonal quadrants in Fig. 4, *B* and *C*). The functionalities of these genes varied from neuropeptides and receptors (*Crh*, *Trh*, *Npy2r*, and *Calca*), complement activation and inflammation (*Serping1*, *C1ql2*, and *A2m*), and extracellular matrix activity (*Lamb3*, *Zic3*, and *Zic4*) (Fig. 4*C*). Additional genes related to cell motility and axonemal activity (*Hydin*, *Dnah12*, and *Rsph9*) were similarly downregulated in the NTS at 12 wk of age along with those whose transient expression was specific to that timepoint (Fig. 2, *D* and *F*).

The set of 314 genes upregulated in the NTS at 10 wk showed minimal overlap in upregulated genes in the RVLM and CVLM beginning at 10 wk of age (Fig. 4*D*). Mapping the 10-wk NTS-specific genes (299 genes) to other timepoints in the RVLM and CVLM revealed that a majority (193 genes) did not show differential regulation in those regions even at other timepoints (Fig. 4*E*, *middle* cell). Of the 314 genes perturbed beginning at 10 wk in the NTS, there were 39 genes whose perturbation began earlier in the RVLM, at 8 wk (Fig. 4*B*). Interestingly, several of these genes were involved in synaptic processes, neurotransmitter transport, and ion channel activity (Fig. 4*F*). Our results suggest that neurotransmitter and synaptic vesicle release processes are perturbed in the RVLM at 8 wk by genes such as *Sv2c* and *Cplx2* (68, 69), which are regulated in the NTS at 10 wk, setting off a cascade of dysregulation in the NTS, recruiting genes such as *Rims1, Syn3, Ncs1*, and *Rph3a*, in similar processes (69–72). Other genes involved in calcium homeostasis, such as *Atp2b3* and ryanodine receptor *Ryr2*, are also upregulated in the NTS at 10 wk while showing no change in the RVLM (73–75). In addition, differential regulation of genes involved in extracellular matrix organization such as collagen-encoding *Col11a2* and *Col4a1* (76), and genes related to cytoskeleton organization influencing synaptic plasticity such as *Ank1* and *Kalrn* (77, 78) was delayed further in the RVLM, not showing upregulation until the chronic hypertensive stage at 24 wk.

### Comparing Dynamic Expression Patterns Between the CVLM and RVLM

**Figure 5.**
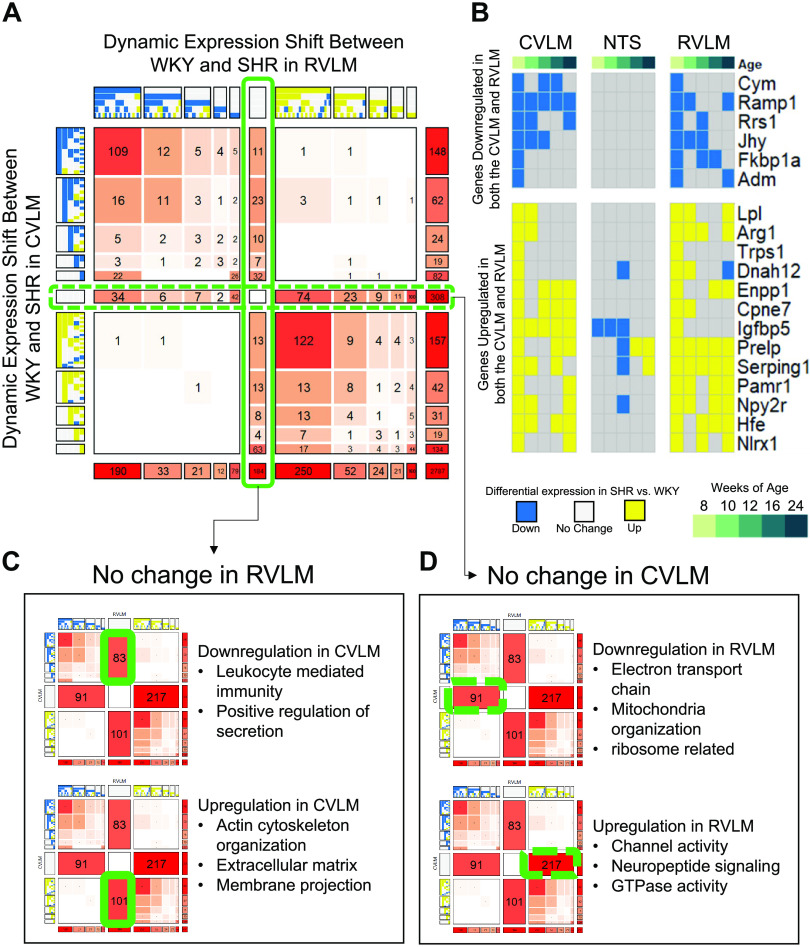
Comparative pattern analysis of spontaneously hypertensive rat (SHR) vs. Wistar-Kyoto (WKY) differential expression dynamics between the caudal ventrolateral medulla (CVLM) and rostral ventrolateral medulla (RVLM). *A*: COMPACT matrix comparing dynamic patterns in the CVLM with those in the RVLM. The solid green line down the center of the matrix represents genes that were not differentially expressed in the RVLM but did show differential expression in the CVLM (C). The dashed green line across the middle of the matrix represents genes that were not differentially expressed in the CVLM but did show differential expression in the RVLM (C). *B*: heatmap showing the discretized patterns of genes that were initially downregulated (top) or upregulated (bottom) in the CVLM and RVLM at 8 wk of age but that were not initially upregulated in the NTS. Each column is representative of the discretized patterns for the given region at each timepoint averaged over multiple biological replicates within each group. *C*: examination of the pathways enriched in genes that showed no change in the RVLM but that were downregulated (*top*) and upregulated (bottom) in the CVLM. *D*: examination of the pathways enriched in genes that showed no change in the CVLM but that were downregulated (*top*) and upregulated (bottom) in the RVLM.

We next looked at the comparison between the RVLM and CVLM for common and distinctive differential gene regulation patterns (Fig. 5*A*). Given their opposing functions in sympathetic drive, it is surprising that at the transcriptional level, there were exceptionally few genes with opposite patterns of dysregulation (antidiagonal quadrants in Fig. 5*A*). Instead, we found that the dominant trend was that of similar dysregulation in both regions, with a high number of genes showing early and persistent down- and upregulation over time (109 and 122 genes, respectively). Of these 231 genes, only 19 genes were not similarly dysregulated in the NTS, further underscoring the theme of systemic differential gene regulation across autonomic circuits in SHRs (Fig. 5*B*). One of the genes, *Ramp1*, is associated with calcitonin-like ligand receptors, which is a highly potent vasoactive peptide that is protective against hypertension, and its early and sustained downregulation in the SHR corresponds to a loss of protection against autonomic dysfunction that primes the system for the subsequent development of hypertension (79). NLRX1 is a member of the NOD-like receptor family and modulates both inflammasome activation and cytokine signaling, although the mechanism and whether it exacerbates or attenuates these responses is highly controversial (80, 81). *Igfbp5* encodes a multifunctional protein that is of particular interest as its dynamic patterns differed across all three autonomic regions (Fig. 5*B*). IGFBP5 has been shown to act as a molecular switch to insulin growth factor signaling, which can modulate neuronal function through altering of excitability and synaptic connections, as well as contribute to neuronal apoptosis and axon degeneration (82–85).

Although the expected level of opposite patterns of gene regulation was not present in the CVLM versus RVLM in SHRs, there were still a number of genes and processes that were dysregulated in only one of these regions (Fig. 5, *C* and *D*). Genes that showed no dysregulation in the RVLM but were downregulated in the CVLM were moderately enriched for processes involved in leukocyte activation and the complement system as well as positive regulation of secretion (*q* < 0.2). Some notable genes encoded for complement proteins C1R and C1S, kainate receptor GRIK1, allograft inflammatory factor AIF1, and integrin subunit ITGB2 (Fig. 5*C*, *top*). Genes that showed upregulation in the CVLM but remained unchanged in the RVLM were enriched for components of the extracellular matrix (*q* < 0.01) and mildly enriched for genes involved in membrane projection (*q* < 0.12) and organization of the cytoskeleton (*q* < 0.15) (Fig. 5*C*, *bottom*). Genes that were downregulated in the RVLM while showing no change in the CVLM were highly enriched for ribosomes, mitochondrial organization, and the electron transport chain (*q* < 1*e*^−4^), with several genes encoding various subunits of the NADH-ubiquinone oxidoreductase complex, also known as complex I of the electron transport chain (Fig. 5*D*, *top*). A much larger number of genes showed upregulation in the RVLM while exhibiting no differential in the CVLM. These genes were enriched for ion channel activity, neuropeptide signaling, and GTPase activity (*q* < 0.05) (Fig. 5*D*, *bottom*).

### Dynamic Differential Regulation of Neuromodulatory Genes in the SHR

**Figure 6.**
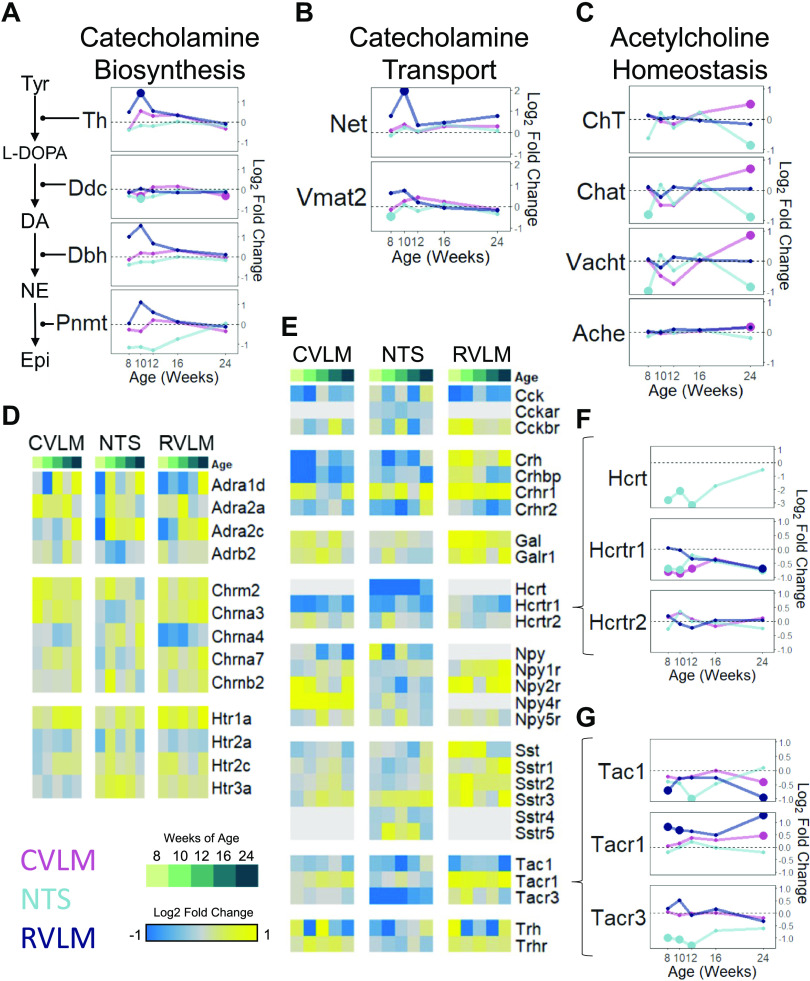
Differential expression dynamics of select neuromodulatory pathways in multiple brainstem regions in spontaneously hypertensive rats (SHRs) compared with Wistar–Kyoto (WKY) rats. *A–C*: differential expression of genes involved in catecholamine biosynthesis (*A*), catecholamine transport (*B*), and acetylcholine homeostasis (*C*) over time. Values represent the pairwise log_2_(fold change) in SHRs compared with WKY rats at each timepoint. Enlarged points represent statistically significant data with *q* < 0.2. *D* and *E*: heatmaps showing the log_2_(fold change) in SHRs with respect to WKY rats for catecholaminergic and cholinergic receptors (*D*) and select neuropeptides and their cognate receptors (*E*). *F* and *G*: differential expression of hypocretin and its cognate receptors (*F*) and tachykinin and its cognate receptors (*G*).

To study how the excitability of neurons in the autonomic brainstem regions is altered in the SHR, we examined the differential gene expression of catecholamine biosynthesis and transport, acetylcholine homeostasis, ion channels, and glutamate and GABA homeostasis and receptors. Below, we highlight gene expression changes that are particularly relevant to the modulation of neuronal function-relevant pathways with a collective impact on the functional state of the brainstem during hypertension development. The most significant changes can be seen in the differential expression of genes encoding TH, the rate-limiting enzyme in catecholamine synthesis (Fig. 6*A*), and NET, the norepinephrine transporter, where they are transiently upregulated in the RVLM at 10 wk of age (*q* < 0.2) (Fig. 6*B*). Similar, although less statistically significant, changes were seen in *Dbh, Pnmt*, and *Vmat2*. On the other hand, genes involved in acetylcholine homeostasis showed distinct patterns in the NTS and CVLM but not the RVLM (Fig. 6*C*). In the NTS, genes encoding CHAT, the enzyme that synthesizes acetylcholine, as well choline and acetylcholine transporters CHT and VACHT, respectively, showed notable downregulation early in hypertension development, at 8 wk of age, and at the extended chronic stage at 24 wk. In contrast, these genes relevant to cholinergic synthesis and transmission showed the opposite pattern in the CVLM compared with the NTS at the extended chronic hypertensive stage (Fig. 6*C*).

As for receptors of both catecholamines, the gene encoding ADRB2, an important receptor for autonomic function, was downregulated in the NTS during the hypertension onset at 10 and 12 wk (Fig. 6*D*). Within the class of genes encoding nicotinic cholinergic receptors, *Chrna7* showed little dysregulation in any of the three brainstem regions, whereas *Chrna3* showed upregulation in both the RVLM and CVLM, while it was transiently downregulated at 10 wk in the NTS. *Chrna4* showed particularly strong downregulation in the RVLM beginning at 8 wk and normalizing around 16 wk. Genes encoding for nicotinic receptor *Chrnb2* and the only muscarinic receptor showing differential expression, *Chrm2,* showed transient upregulation in the NTS at 10 wk (Fig. 6*D*).

We examined the differential gene expression of select neuropeptides and their cognate receptors (Fig. 6*E*). Corticotropin-releasing hormone (*Crh*), thyrotropin-releasing hormone (*Trh*), and Npy receptor 2 (*Npy2r*) all displayed distinct patterns in the NTS versus the RVLM (Fig. 4*C*). Hypocretin (*Hcrt*), also known as orexin, which was detectable only in the NTS in the present data set, was downregulated in SHR versus WKY rats at the hypertension onset and showed a trend toward normalization by 24 wk (Fig. 6*F*). Orexin has been shown to increase vagal activity to the heart (86). The substantial decrease in expression in the NTS was therefore consistent with previous results showing decreased vagal tone and increased sympathoexcitation seen in the SHR (87). *Hcrtr1* was downregulated in all three brainstem regions, with more significant changes in the NTS and RVLM at earlier timepoints and in all three regions at the chronic stages (Fig. 6*F*). *Tac1*, which encodes the precursor protein for substance P and is associated with multiple autonomic functions (88), was downregulated in all three brainstem regions in a sequence that mirrored the transcriptome-wide patterns: downregulation began in the RVLM at 8 wk, shifted to the NTS during onset, and was then downregulated in the RVLM and CVLM at the chronic hypertensive stage (Fig. 6*G*). Of the TAC1 receptor genes, *Tacr1* was upregulated in the RVLM, whereas *Tacr3* was downregulated in the NTS, with patterns for both receptors largely persisting regardless of developmental stage, although with varying levels of statistical significance (Fig. 6*G*).

**Figure 7.**
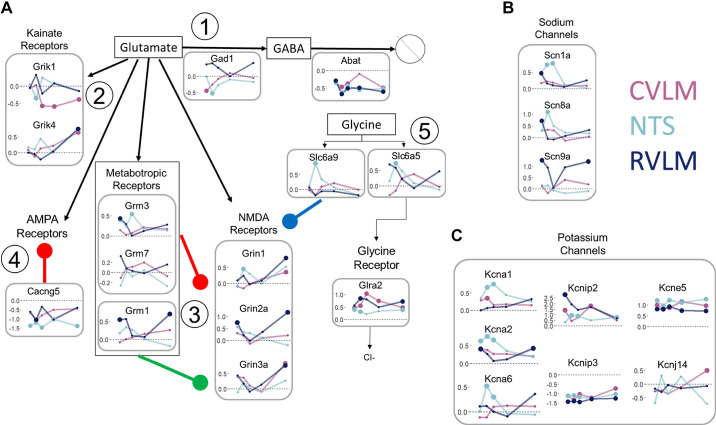
Region-dependent differential gene expression of excitatory and inhibitory synaptic processes in spontaneously hypertensive rats (SHR) vs. Wistar–Kyoto (WKY) rats. *A*: traces of differentially expressed genes involved in the metabolism and signaling of glutamate, GABA, and glycine. *1*) glutamate is converted to GABA through the actions of glutamate decarboxylase (*Gad1*). GABA is degraded by GABA transaminase or aminobutyrate aminotransferase (*Abat*). *2*) glutamate activates three different types of ionotropic receptors, kainate, NMDA, and AMPA receptors, and G protein-coupled metabotropic receptors. *3*) *Cacng5*, a Ca^2+^ channel that is also a type II transmembrane AMPA receptor regulatory protein that inhibits the activity of AMPA receptors. *4*) metabotropic receptors can have both excitatory and inhibitory action on NMDA receptors; *Grm1* enhances the activity of NMDA receptors, whereas *Grm3* and *Grm7* inhibit the action of NMDA receptors. *5*) *Slc6a9* encodes the glycine transporter Glyt1, which removes glycine from the synaptic cleft and regulates glycine levels involved in neurotransmission mediated by NMDA receptors. *Slc6a5* encodes glycine transporter Glyt2, which facilitates inhibitory transmission through glycine release. Glycine activates ligand-gated Cl^−^ channel *Glra2*, which downregulates neuronal excitability. *B*: dysregulated Na^+^ channels. C: dysregulated K^+^ channels. All plots reflect log_2_(fold change) on the *y*-axis as in Fig. 6. The dashed line in each plot represents no change in expression, with points above (below) the line representing an increase (decrease) of expression in SHRs compared with WKY rats. From left to right, points represent 8, 10, 12, 16, and 24 wk of age. Enlarged points represent statistically significant data with *q* < 0.2.

We examined the differential gene regulation of the glutamate, GABA, and glycine transmitter systems. *Gad1*, responsible for the synthesis of GABA from glutamate, was particularly downregulated at 10 wk in the NTS, whereas *Abat*, responsible for the degradation of GABA, was downregulated in all three regions (Figure 7*A1*). Among the glutamate receptors, the *Grik1* gene stood out as being particularly downregulated in the CVLM during and after the onset of hypertension. Receptor genes *Grik4, Grin1, Grin2a*, and *Grin3a* and metabotropic glutamate receptor gene *Grm1* all showed upregulation in the RVLM at the stage of extended chronic hypertension, consistent with overexcitability in the RVLM leading to higher sympathetic output (Figure 7*A2*). *Grin2a* and *Grm1* additionally showed upregulation at 8 wk along with other metabotropic glutamate receptors *Grm3* and to some extent *Grm7*, which are known to have opposite influences on NMDA receptor function (Figure 7*A3*). In addition, *Cacng5* functions as a modulator of AMPA receptors, and its systemic downregulation was indicative of decreased glutamate receptor deactivation and increased potency (Figure 7*A4*) (89, 90). Interestingly, glycine transporters SLC6A5 and SLC6A9 act at excitatory and inhibitory synapses, respectively (91), and the corresponding genes showed upregulation in the NTS at 10 wk, whereas the gene encoding glycine receptor GLRA2, a ligand-gated Cl^-^ channel contributing to the generation of inhibitory synaptic currents, was systematically upregulated (Figure 7*A5*). With regard to genes encoding Na^+^ channels, *Scn9a* was persistently upregulated in the RVLM with the exception of 12 wk, whereas *Scn8a* and *Scn1a* appeared to show upregulation in the NTS around the onset of hypertension at 10 and 12 wk (Fig. 7*B*). The genes encoding Shaker-related K^+^ channels *Kcna1*, *Kcna2*, and *Kcna6* were all upregulated in the NTS during 10–12 wk, whereas genes encoding K^+^ channel-interacting proteins *Kcnip2* and *Kcnip3* were systemically up- and downregulated, respectively (Fig. 7*C*). The gene encoding K^+^ channel *Kcne5* was systemically upregulated, whereas *Kcnj14*, an inward rectifying K^+^ channel gene, was highly correlated with genes involved in acetylcholine homeostasis in all three regions, with the most prominent pattern being downregulation in the NTS at 8 and 24 wk, albeit at lower levels of significance (Fig. 7*C*).

Taken together, our results show that coordinated gene expression changes occur in pathways related to catecholamine and acetylcholine homeostasis, ion channels, and glutamate and GABA receptor homeostasis with a collective impact on multiple neuronal functions during the development of hypertension. The early dynamics are characterized by the downregulation of vagal cholinergic pathways in the NTS and the upregulation of catecholaminergic pathways in the RVLM. The later changes also contain modulation of glutamatergic and GABAergic pathways as well as multiple ion channels altering the neuronal excitability. Thus, the differential gene expression patterns of several neuropeptides, ion channels, and receptors indicate an emerging dynamic of imbalance between sympathoexcitation and sympathoinhibitory mechanisms within the autonomic brainstem regions in the SHR.

## DISCUSSION

Here, we provide new time series transcriptomic data on the changes in multiple brainstem autonomic regions during the development of hypertension. We analyzed the multifactorial data using an unbiased comparative pattern analysis to extract dominant and subtle as well as regionally and temporally localized trends in the data. Overall, there appear to be large systemic changes that occur throughout the brainstem autonomic regions, regardless of the stage in hypertension development in the female SHR compared with WKY rats. With this systemic dysregulation as a background, a cascade of transcriptional changes is initiated in the RVLM at 8 wk of age, with many of these genes dysregulated in the NTS beginning at 10 wk. At this stage of the onset of hypertension (10 wk), upregulation of genes in the NTS largely dominates the change in the transcriptomic landscape across all three brainstem regions. A key feature at 12 wk was an upregulation of genes in WKY rats in the NTS, which was not observed in the NTS in SHRs. Although there were minimal transcriptomic differences in SHRs versus WKY rats at 16 wk across the three brainstem regions, there is an increase in the number of dysregulated genes in the chronic persistent stage of hypertension at 24 wk, particularly in the RVLM and CVLM. We focused on neuromodulatory processes including neuropeptides, catecholaminergic and cholinergic pathways, ion channels, and synaptic processes, to localize the dynamic dysregulation in SHRs to key components over time with a likely impact on excitability of brainstem autonomic neurons in a region- and stage-specific manner during hypertension development. The multiregion time series transcriptomic data set can serve as a resource for the community to help explore the wide range of neural molecular systems dysregulated in hypertension.

Our results uncovered a dominant signature of gene expression changes that are consistent across brainstem autonomic regions and persistent throughout the development of hypertension. Interestingly, these systemically dysregulated genes were highly enriched for processes related to the activation of the immune system, antigen processing and presentation, and the complement system. Some notable genes in these processes are the rat isoforms of the human major histocompatibility complex (MHC; the *RT1* family), responsible for the presentation of antigens on the cell surface, as well as one of its receptors, PirB (*Lilrb3a*). The need for functional MHC I for proper neuronal function has been demonstrated (92), while the PirB receptor has been shown to be expressed in the central nervous system, particularly in highly plastic regions with an impact on myelin regeneration and axon guidance (93–96). Indeed, MHC I has been shown to influence the excitability of neurons, specifically influencing NMDA receptor function and trafficking of AMPA receptors (49–51). The association of MHC I with the PirB receptor as well as cross talk with the complement system has been implicated in the pruning of neural connections (93, 96). The systemic dysregulation of these genes in the autonomic nuclei of hypertensive rats sheds more light on the expected connection between the immune system, neuronal function, and hypertension development (97–103). Follow-up work is needed to elucidate the mechanisms of influence of these highly polymorphic genes where the exact function of the different isoforms is still unclear (93).

Beneath the systemic dysregulation throughout the brainstem, our analysis uncovered a cascade of dynamic changes, beginning in the RVLM at the prehypertensive stage and shifting to the NTS during the hypertension onset. Although there is little transient gene regulation once hypertension has developed at 16 wk of age, there is an increase in gene dysregulation in both the RVLM and CVLM at the extended chronic hypertensive stage at 24 wk. This reduction in the number of DEGs at 16 wk of age and a resurgence at 24 wk suggests intriguing possibilities. One hypothesis is that 16 wk of age corresponds to a transient plateau in the regulatory changes that are driven by early age-dependent mechanisms driving hypertension and the later gene regulatory changes may be driven by pathological changes that are occurring systemwide in multiple organs, driven by sustained high blood pressure. Our previous study supports this expectation to a certain extent as we found multiple hypertension-relevant pathways dysregulated in the kidney, left ventricle of the heart, adrenal gland, and liver (104). Most interesting is the shift of differential gene regulation from the RVLM to the NTS as hypertension develops. Although the NTS is the integrative processing center of the autonomic circuit, it is the RVLM, the site of sympathetic outflow, that initiates the cascade of dysregulation, which then shifts to the NTS at the onset stage. This pattern was consistent in restrictive analysis with a twofold change threshold as well as in the dynamic pattern analysis that considered lower fold changes.

Our transcriptomic analysis shows that the early hypertension onset stage of 10 wk is marked by a large set of dysregulated genes that is highly specific to the NTS. These genes are enriched for processes such as synaptic function, neuropeptide signaling, and GTPase activity. Modulation of exocytosis and endocytosis at the synaptic terminal by synaptic vesicle trafficking genes and their subsequent impact on the release of neuromodulators such as glutamate, GABA, and other neuropeptides have obvious implications for neuronal excitability (73, 105, 106). Transcriptomic profiles of synaptic communication patterns have been shown to differentiate between neuronal subtypes as have combinatorial patterns of neuropeptides and their cognate receptors (69, 107). Some synaptic genes have been linked to blood pressure maintenance through their regulation of catecholamine release, while many others have additionally been shown to regulate axonal regeneration and neuronal plasticity (68, 70–72). The dysregulation of these processes in the NTS of SHRs at the hypertension onset stage further emphasizes the importance of the NTS as the critical integrative center for autonomic processing. The specific temporal dysregulation of these genes at 10 wk lays the groundwork for follow-up studies that focus on alterations in synaptic activity in the NTS at this stage.

Although the number of genes dysregulated in the NTS at 12 wk is substantially lower than at 10 wk, these genes share a similar profile of distinct upregulation in the WKY rat whereas the temporal profile in the SHR remains unchanged. This lack of upregulation in the NTS in SHRs at 12 wk suggests a loss of function activity. These genes are enriched for processes related to cell motility and cell migration. Neuronal migration is crucial during development as it guides the extension of axonal growth cones (108). Aside from cell motility, primary cilia have been shown to be expressed near ventricles and survey the cerebrospinal fluid for extracellular peptides and thus influence neuronal signaling in a nonsynaptic manner (64). Although nonsynaptic signaling through primary cilia has been mostly noted in the forebrain regions to date, these data emphasize their importance in regions of the brainstem as well, especially as the NTS is in close proximity to the IV ventricle (63). In addition, several neurotransmitter receptors and ion channels have been shown to be expressed on the membranes of primary cilia that influence a variety of signaling pathways and regulation of cellular processes through calcium signaling (109, 110). The lack of upregulation of these processes in the SHR NTS at the hypertension onset age of 12 wk needs follow-up mechanistic analysis.

Altough the transient dysregulation at 24 wk shows a balance between the three regions at a high fold change threshold (>2), dynamic pattern analysis considering smaller gene expression changes shows that changes in the RVLM and CVLM far outweigh those in the NTS. The autonomic control regions of the RVLM and CVLM are known to exert opposing functions at the physiological level (4, 15, 16, 18–21). Considering the circuit function of these regions, the lack of opposing gene expression patterns between the RVLM and CVLM observed in our transcriptomic analysis is intriguing. When we examine the genes that are similarly regulated between the two regions, a large majority are similarly dysregulated in the NTS as well, further underscoring the dominant theme of systemic dysregulation regardless of the hypertension developmental stage in the SHR. Several genes were differentially regulated in the SHR in either the CVLM or RVLM but not the other at multiple stages of hypertension development. Among these gene sets, the downregulation of genes involved in the electron transport chain and mitochondrial processes and the upregulation of genes involved in neuropeptide signaling and GTPase activity in the RVLM warrant further investigation. Dysregulation of oxidative phosphorylation leading to oxidative stress is a well-documented source of aberrant sympathetic drive (111–113) and such a large increase of genes involved in peptide signaling and GTPase activity points to additional mechanisms driving increased excitability in the RVLM in SHRs.

When we examine catecholaminergic and cholinergic processes, the sequence of dysregulation in the brainstem differs from the overall transcriptomic patterns outlined above. Instead, it is the NTS with downregulated expression of genes involved in acetylcholine homeostasis early on at 8 wk of age and the RVLM that showed an increase in catecholamine biosynthesis genes at the hypertension onset period of 10 wk. Acetylcholine has been reported to promote sympathoinhibition in the NTS (114, 115). The observed downregulation of genes involved in acetylcholine synthesis and transport before the development of hypertension at 8 wk would indicate an attenuation of sympathoinhibition at this stage, likely tipping the balance of sympathetic activity in the autonomic blood pressure control circuits toward sympathoexcitation. Our results suggest that the initial dysregulation of cholinergic systems in the NTS is shifted toward altered catecholaminergic processes in the RVLM at the early hypertension onset stage. Although it has been well established that catecholaminergic drive is increased in the RVLM of hypertensive rats at various stages in the development of hypertension (29, 30, 116, 117), our transcriptomic analysis uncovered a transient increase taking place at the hypertension onset, 10 wk of age, and normalizing afterward. Our transcriptomics results on transient dynamics, while consistent with an increase in catecholaminergic drive from RVLM, point out a nuanced regulation in which temporally restricted dysregulation of gene expression precedes and is associated with persistent changes at the protein and functional level. The significance and extent of dysregulation of catecholaminergic genes appear to be specific to the RVLM. In contrast, multiple studies have shown decreased catecholaminergic drive in the NTS in the SHR, pointing to the restraining influence of A2 neurons on the blood pressure set point and variability (118–122). The gene expression changes corresponding to the catecholamine biosynthesis processes localized to the A2 subpopulation of neurons were not detectable in the present transcriptomic analysis at the whole NTS level.

Our analysis uncovered an interesting array of differential gene expression in ion channels that points to a shift in the balance between excitatory and inhibitory activity in the three brainstem regions. Both Na^+^ and K^+^ channels were upregulated at earlier timepoints. Shaker-related voltage-gated K^+^ channels *Kcna1, Kcna2*, and *Kcna6* encode members of the Kv1 family and have been shown to be present in multiple subcellular locations (123). When present at the presynaptic terminal, they have been shown to reduce the excitability of the nerve terminal and prevent aberrant firing and transmitter release (124). If the NTS presynaptic neurons in the SHR overexpressing these genes are projecting to the CVLM, their lack of activation could result in less inhibition of the RVLM by the CVLM. Members of the *Kcnip* gene family encode Ca^2+^-sensing K^+^-interacting proteins that modulate the A-type current generated from the Kv4 (Shal) family of K^+^ channels. When associated with members of the *Kcnip* family, Kv4 K^+^ channels inactivate slower and have a faster recovery from inactivation (125). There are also examples of *Kcnip* family members inducing transcriptional repression and influencing intracellular Ca^2+^ concentration (126). Although there is evidence that the functions of different *Kcnip* family members are distinct in hippocampal neurons (127), the meaning of the opposite and persistent dysregulation of *Kcnip2* and *Kcnip3* in the brainstem autonomic regions needs to be explored in the SHR.

Our results point to a shift in gene expression in the glutamatergic and GABAergic systems within the brainstem regions during hypertension development. For example, the downregulation of *Abat*, involved in the degradation of GABA, in conjunction with the upregulation of glycine receptor *Glra2* suggests a systemic increase in inhibitory signals in SHRs. This observation is further supported by the downregulation of excitatory receptor gene *Grik1* in the CVLM. Several NMDA receptors are transiently upregulated in the RVLM at the prehypertensive stage and are then marked by a resurgence later at the extended chronic stage at 24 wk. G_i_-coupled *Grm3* and G_q_-coupled *Grm1* follow a similar pattern and with the inhibition and activation of NMDA receptors, respectively, the combined effect of the concurrent upregulation of these genes in the RVLM is difficult to predict. Interestingly, while AMPA receptors show no detectable differential gene expression between the SHR and WKY rat, voltage-gated Ca^2+^ channel gene *Cacng5*, also known as a type II AMPA receptor regulatory protein, is persistently downregulated in all three regions. Less inhibition of AMPA receptors by CACNG5 would indicate increased excitability of these regions (90), which is largely in contrast to the evidence of increased inhibitory signaling from the respective down- and upregulation of *Abat* and glycine receptor gene *Glra2*. In fact, recent data have further highlighted the importance of glycine in inhibitory signaling, suggesting that glycine influences the strength of inhibition while GABA is responsible for the threshold of excitability (128). Other studies have suggested that glycinergic signaling helps mediate the sympathoinhibition of the RVLM by the CVLM (129). Therefore, the coordination of GABA and glycine seems of particular importance given the concurrent dysregulation of both these genes throughout the brainstem. Interestingly, the glycine transporters *Slc6a9* (GlyT1), which acts at both pre- and postsynaptically at excitatory synapses to modulate the glutamatergic action on NMDA receptors, and *Slc6a5* (GlyT2), which acts presynaptically at inhibitory synapses, are both upregulated in the NTS around the onset of hypertension (91, 130, 131). This transient increase in expression is indicative of increased inhibitory signals and decreased excitatory signals in the NTS. A decrease in excitatory signaling to the CVLM around the onset could also result in less inhibition of the RVLM, collectively leading to a higher sympathetic drive towards hypertension in SHRs.

This work represents a comprehensive characterization of the broad dynamic transcriptomic changes taking place in three autonomic nuclei throughout the development of hypertension. Assaying transcriptional changes between SHRs and WKY rats beginning before and throughout the development of the hypertensive phenotype through to a stage of chronic hypertension allowed us to investigate pathways previously studied in the chronic hypertensive condition and interpret the changes in a dynamic context as an evolving multiregion control system in the brainstem. We further uncovered both persistent dysregulation across all three regions as well as a cascade of temporal changes beginning in the RVLM and shifting to the NTS at the stage of hypertension onset. These transient transcriptomic changes likely shift the overall system towards a new set of intra- and intercellular interactions within the brainstem nuclei as well as across the autonomic control circuit. The results on enriched functional processes uncovered here serve as a starting point for follow-up studies aimed at further interrogating the mechanisms of their influence on the initiation and progression of hypertension. A transcriptomics time series at high temporal resolution with matched physiological measures can further inform on the dynamic relationship between the molecular changes and their functional impact on blood pressure physiology. In that regard, our time series transcriptomic data set of brainstem autonomic nuclei is a step in that direction as a unique resource that can aid in the exploration of the wide range of neuronal molecular systems and control circuits that drive autonomic dysfunction.

## DATA AVAILABILITY

The time series RNA-seq paired-end data are available through the National Center for Biotechnology Information Gene Expression Omnibus and Sequence Read Archive under Accession No. GSE234784.

## GRANTS

This work was supported by National Institutes of Health Grants U01HL133360 and R01HL161696 (to J.S.S. and R.V.) and National Institutes of Health Common Fund SPARC program OT2OD030534 (to R.V.).

## DISCLAIMERS

The funders had no role in the study design and implementation, interpretation of the results, and preparation of the manuscript.

## DISCLOSURES

No conflicts of interest, financial or otherwise, are declared by the authors.

## AUTHOR CONTRIBUTIONS

J.S.S. and R.V. conceived and designed research; A.M. and A.S. performed experiments; A.M., L.K., A.S., and R.V. analyzed data; A.M., L.K., A.S., J.S.S., and R.V. interpreted results of experiments; A.M., L.K., A.S., and R.V. prepared figures; A.M., L.K., A.S., and R.V. drafted manuscript; A.M., L.K., A.S., J.S.S., and R.V. edited and revised manuscript; A.M., L.K., A.S., J.S.S., and R.V. approved final version of manuscript.
